# Cleaning the palate and tongue without nausea: a mixed methods study exploring the appropriate depth and direction of oral care

**DOI:** 10.1186/s12903-021-01414-5

**Published:** 2021-02-12

**Authors:** Yang Cheng, Yu-feng Zhou, Ya-ping Ding, Ying Xing, Enfang Shan, Hang Sun

**Affiliations:** School of Nursing, Nanjing Medical University, 140 Hanzhong Road, Nanjing, 210000 Jiangsu People’s Republic of China

**Keywords:** Oral care, Oral cleaning depth, Oral cleaning direction, Non-nausea

## Abstract

**Background:**

It is advisable to clean the palate and tongue thoroughly during oral care to protect against nosocomial infections. However, improper cleaning may cause nausea. To date, no robust data are available regarding how to implement this procedure properly. Furthermore, traditional cotton balls, forceps and normal saline are still used in clinical in China. This mixed methods study aimed to explore the appropriate depth and direction of cleaning methods for palates and tongues without causing nausea and the factors influencing cleaning depth and discomfort in traditional oral care.

**Methods:**

Our study recruited students (n = 276) from a medical university. The first phase was a quantitative study, in which forceps were slowly inserted into their throats until the gag reflex was triggered, and then, the insertion depth was measured. After that, participants were randomly divided into two groups. In group A, palates and tongues were cleaned coronally and then sagittally, with the converse order used for group B. The extent of nausea was measured. Additionally, the qualitative data were types of discomfort other than nausea reported by the participants.

**Results:**

The tolerable depths (without causing nausea) for cleaning the palate and tongue were 6.75 ± 1.07 cm and 6.92 ± 1.11 cm, respectively. Participants of male sex and with high BMI (overweight/obese) were associated with greater tolerable cleaning depth. The extent of nausea caused by cleaning both the palate and the tongue sagittally was higher than that elicited by coronal cleaning (*p* = 0.025 and *p* = 0.003, respectively). Other discomforts included itching, saltiness and coldness.

**Conclusion:**

It is appropriate to increase the cleaning depth of the palate and tongue for adult males and overweight/obese individuals. Moreover, coronal cleaning causes lower levels of nausea, and traditional oral care appliances should be improved.

## Background

Oral health profoundly affects general health [1]. One of the effective approaches to maintain oral health is oral care, which has been evidenced to maintain oral health by reducing bacteria in the oral cavity [2]. As routine care, oral care is an effective measure to reduce the risk for infection [3], decrease the incidence of pneumonia [4], prevent the occurrence of mucositis [5] and significantly improve the quality of life of the patient [3]. It has been reported that approximately one in ten cases of death caused by pneumonia in residents of nursing homes for the elderly could be prevented by improving oral hygiene [6]. However, providing oral care deeply or improperly may cause nausea, which is associated with the gag reflex.

**Fig. 1:**
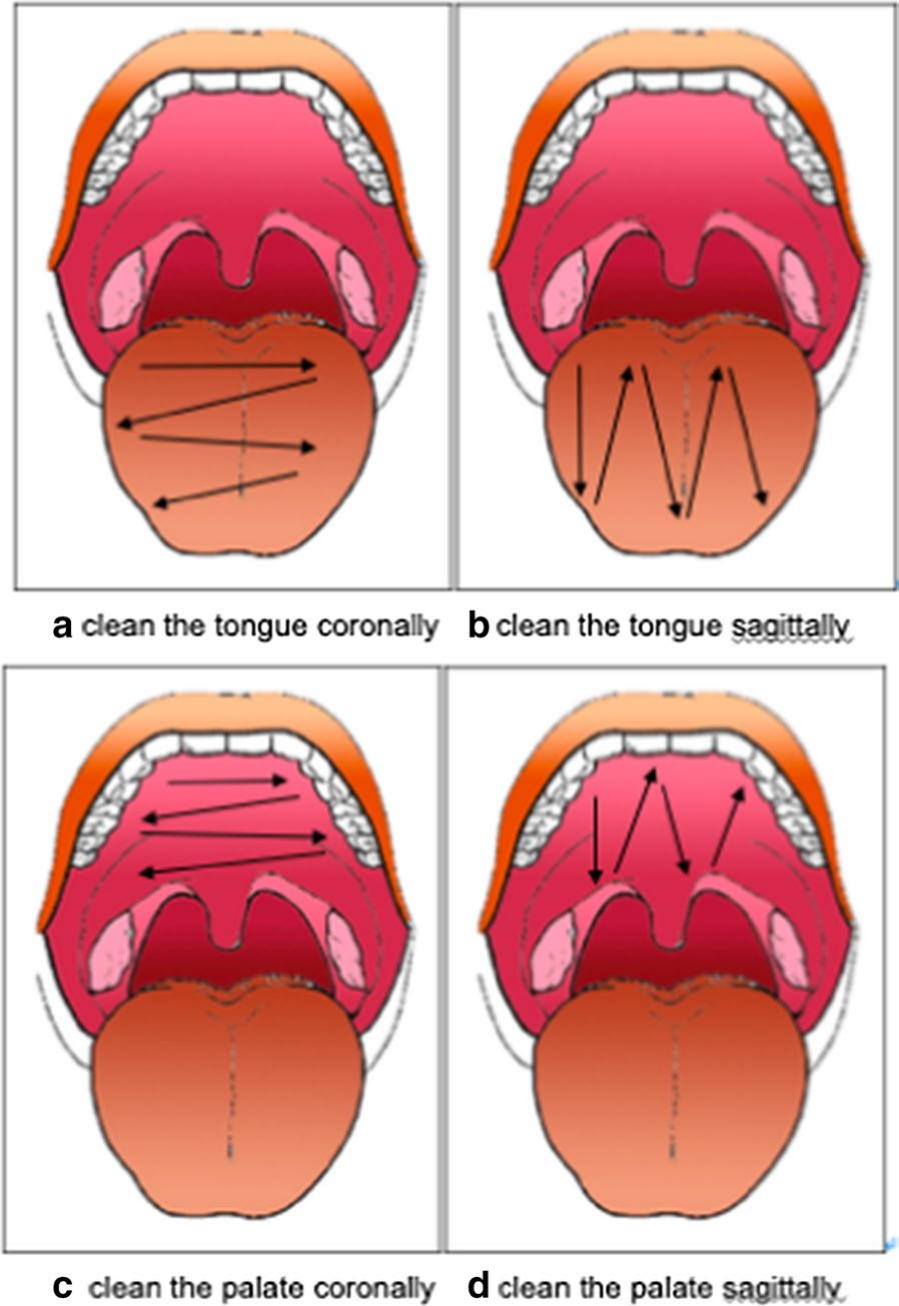
Cleaning the oral cavity sagittally and coronally

Nausea is a physiological defence reflex [1], and the prevalence of self-reported gagging during dental treatment is 8.2% [7]. Frequent gagging is even related to correlative fear [1] and hinders the receipt of adequate dental care [8]. Many fixed areas can trigger the gag reflex, commonly including the faucial pillars, the base of the tongue, and the soft palate, uvula, and posterior pharyngeal wall [9]. Since there are fixed areas that can trigger the gag reflex, and tongue cleaning has been recommended for the improvement of oral health [10], it is reasonable to measure the depth at which this response is triggered, which could help to avoid the gag reflex caused by oral care and keep the mouth as clean as possible. However, there is limited literature that specifically addresses the depth of oral cleaning [11] and insufficient evidence to date on factors influencing gag reflex sensitivity, such as sex [12]. In addition, the hard palate and tongue cannot be covered by a single cotton ball in one step due to their width; thus, they require repeated back and forth movements during cleaning. There are two ways of cleaning, as illustrated in Fig. 1: sagittally and coronally; however, which of these is less likely to cause nausea in patients has not been reported to date.

The purpose of this mixed method study is to investigate oral care methods, including appropriate cleaning depth and direction without nausea, and to illuminate the factors related to the appropriate cleaning depth and discomfort of traditional oral care. Current research only considers the oral hygiene effect but neglects cleaning methods, and this study attempted to fill this gap.

## Methods

### Study design

This was a mixed methods study that included two parts: quantitative research and qualitative research [13].

### Participants

The sample size was estimated using the formula ${n=\left({t}_{\alpha /2}S/\delta \right)}^{2}$ [14]. A total of 266 subjects were calculated based on the values of S = 1, *δ* = 0.12, and α = 0.05. Ultimately, 276 sophomores (mean age = 19.63 ± 0.75 years; age range = 18–22 years; 42 men) were recruited from a school (mean height = 163.82 ± 6.80 cm; height range = 149.5–189 cm; mean weight = 57.77 ± 10.09 kg, weight range = 39–130 kg). All participants were informed about what their involvement would consist of and the objectives of this study.

The inclusion criteria were good health, good oral hygiene, and voluntary participation; there were no exclusion criteria in this study. Prior to the study, this research was approved by the Ethics Committee at the School of Nursing, Nanjing Medical University, China (2020-SR-146).

## Experimental procedure

### Preparation

The experiment began two hours after participants had eaten a meal, and all basic information and data were recorded by investigators. To identify the factors influencing cleaning depth without causing nausea, demographic characteristics, such as age, sex, height, and weight (using HNH-219 Type; Omron), were collected first. The errors of height and weight were in the 0.1 cm and 0.1 kg ranges, respectively. Body mass index (BMI) was calculated as weight divided by height squared (kg/m^2^). Overweight and obesity, normal weight and underweight were defined as BMI values > 24, 18.5–24, and < 18.5, respectively.

Three researchers were trained to implement the research. In this study, disposable plastic forceps (length, from shaft knot to tip, 15 cm), dry cotton balls (diameter, 3 cm), and disposable sterile dressing kits (using Deyi Kang A-10) were used to measure the cleaning depths of the palate and tongue without causing nausea. Cotton balls were soaked in 0.9% saline solution and squeezed until they were half-dry. The diameter of the wet cotton balls was approximately 1.5 cm after being squeezed, and the forceps held the centre of the wet cotton ball. Participants were asked to open their mouth so that there were two finger widths between the upper and lower incisors (open mouth moderately).

### Measurement of cleaning depths of the palate and tongue without causing nausea

To measure the greatest tolerable cleaning depth for the palate, forceps with a saline cotton ball were inserted into the participant’s mouth at the maxillary central incisor and guided slowly down to the throat along the palate. When the participants could no longer tolerate the insertion or the gag reflex was triggered, they would inform the examiner by raising their hands. The examiner marked the forceps at the position of the maxillary central incisor and then removed the forceps from the participants’ mouths. The insertion distance of the forceps from the cotton ball to the mark was measured using a mm ruler. The mean value of three measurements was recorded as the cleaning depth for the palate without causing nausea. A similar procedure was followed to measure the depth at which the tongue could be cleaned.

### Measurement of nausea extent caused by coronal and sagittal cleaning

A randomized crossover trial was designed to measure the extent of nausea caused by cleaning the palate and tongue coronally and sagittally. Notes with groups were concealed in an opaque envelope, and participants were randomly grouped by drawing lots before the measurement. A total of 134 participants were allocated to group A and 142 to group B. In group A, participants’ palates and tongues were first cleaned coronally and then sagittally within their tolerable cleaning depth. Conversely, participants in group B were first cleaned sagittally and then coronally. All participants’ palates were cleaned first, followed by their tongues. After cleaning, the participants rated the extent of nausea they felt using a visual analogue scale (VAS) [15], which ranged from 0 (not at all) to 10 (very strong). To rule out the effect of cleaning time on nausea, we measured it using a stopwatch from mouth opening to mouth closing.

### Recording of other discomforts during the cleaning of the palate and tongue

To evaluate the comfort of Chinese traditional oral care tools and the solutions used, participants were asked to write down other discomforts they felt during cleaning in an open-ended question: What other discomforts do you feel besides nausea? Summative content analysis [16], an approach used to identify and quantify key words by rereading the text, was implemented independently by two researchers.

### Statistical analysis

The data regarding cleaning depth without causing nausea were near normally distributed and were, therefore, described as the means and standard deviation. Data regarding nausea extent are described as medians and interquartile ranges, as they were not normally distributed. The independent t-test, ANOVA, and multiple linear regression were used to assess differences in cleaning depth without causing nausea (both palate and tongue) between the sexes, and according to height and BMI, pairwise comparisons between these groups were evaluated by an LSD test. Height was stratified according to the median height. The Wilcoxon rank-sum test was used to compare the two directions of palate and tongue cleaning (sagittally and coronally). All data analyses were conducted using IBM SPSS version 22.0 (IBM Corp., Armonk, NY, USA). The *p* < 0.05 was considered statistically significant.

## Results

### Participants’ characteristics

We recruited 276 participants, and their characteristics are summarized in Table 1.

**Table 1:** Characteristics of participants (N = 276)

Characteristics	n (%)
*Sex*	
Male	42 (15)
Female	234 (85)
*Height*	
≥ 163.00 cm	147 (53)
< 163.00 cm	128 (46)
Missing values	1 (0.4)
*BMI*	
Overweight and obese	39 (14)
Normal	212 (77)
Underweight	23 (8)
Missing values	2 (0.7)

### Depth of palate cleaning without causing nausea

The mean depth at which the palate could be cleaned without causing nausea was 6.75 ± 1.07 cm. Further results of univariate analyses are presented in Table 2. There was a significant difference in cleaning depth between males and females, with that for males being deeper than that for females. The univariate analysis also identified a significant difference according to BMI; as BMI increased, the cleaning depth of the palate increased. There were also significant differences among the three measurements (first, second, and third), with pairwise comparisons, followed by ANOVA and LSD tests; all were statistically significant. There was a weak positive correlation between height and mean cleaning palate cleaning depth (*r* = 0.150; *p* = 0.013); however, the difference in the cleaning depth of the palate between participants of different heights was not significant. A multiple linear regression model was used to evaluate the relationships among depth of palate cleaning and sex or BMI, and the results indicated statistical significance (*F* = 9.688; *p* < 0.001) (Table 3).

**Table 2:** Factors influencing the depth of cleaning of the tongue and palate without causing nausea: univariate analysis (N = 276)

Stratification factors	Palate			Tongue		
	Cleaning depth (cm)	*t/F*	*P*-value	Cleaning depth (cm)	*t/F*	*P-*value
*Sex*						
Male	7.30 ± 1.50	− 2.698^a^	0.010	7.45 ± 1.50	− 2.569^a^	0.013
Female	6.65 ± 0.95			6.83 ± 1.00		
*Height*						
≥ 163.00 cm	6.83 ± 1.20	1.405^a^	0.161	6.93 ± 1.28	0.189^a^	0.850
< 163.00 cm	6.65 ± 0.89			6.91 ± 0.88		
*BMI*						
Overweight and obese	7.19 ± 1.36	5.406^b^	0.005	7.37 ± 1.10		
Normal	6.72 ± 1.00			6.90 ± 1.11	6.104^b^	0.003
Underweight	6.32 ± 0.87			6.38 ± 0.93		
*Measuring time*						
1st measurement	6.44 ± 1.19	104.035^b^	< 0.001	6.76 ± 1.21		
2nd measurement	6.77 ± 1.10			6.96 ± 1.15	33.019^b^	< 0.001
3rd measurement	7.03 ± 1.12			7.06 ± 1.17		

^a^t

^b^*F*

**Table 3:** Factors influencing the depth at which the tongue and palate can be cleaned without causing nausea: multiple linear regression (N = 276)

Variable	Palate					Tongue				
	β	Standard error	Standardized regression coefficient	*t*	*P*-value	β	Standard error	Standardized regression coefficient	*t*	*P*-value
Constant	5.642	0.310		17.620	< 0.001	5.563	0.325		17.096	< 0.001
Sex	0.525	0.183	0.176	2.877	0.004	0.394	0.188	0.155	2.535	0.012
BMI	0.334	0.138	0.148	2.420	0.016	0.477	0.145	0.167	2.723	0.007

### Depth of tongue cleaning without causing nausea

The depth at which the tongue could be cleaned without causing nausea was 6.92 ± 1.11 cm. The results of further univariate analyses are presented in Table 2. Similar to the results for the palate, differences between tongue cleaning depth and sex, BMI or measuring time were all statistically significant. Again, there was no statistically significant difference between participants of different heights. Multiple linear regression analysis showed that sex and BMI were associated with mean cleaning depth (*F* = 9.449, *P* < 0.001). The results of further multiple linear regression analyses are shown in Table 3.

### Extent of nausea caused by the two palate cleaning directions

The degrees of nausea caused by cleaning the palate coronally and vertically, according to VAS score, were 1 (0–2) and 1 (0–3), respectively. The extent of nausea caused by sagittal cleaning was significantly higher than that caused by coronal cleaning (Wilcoxon rank-sum test: *Z* = − 2.248; *p* = 0.025). The scores for feelings of nausea for participants in both Groups A and B, where the two directions of cleaning were tested in a different order for each group, are shown in Table 4 and demonstrate that the extent of nausea caused by cleaning the palate was not influenced by cleaning order. Although the difference in the duration of palate cleaning between the two groups was significant, there was no clinical significance. The results of stratified analysis showed that the extent of nausea caused by sagittal cleaning was significantly higher than that elicited by coronal cleaning for females, participants with lower than median height, and participants with normal BMI (Table 5).

**Table 4:** Nausea extent and cleaning time for the two directions for cleaning the palate and tongue

	Palate			Tongue		
	Extent of nausea	*Z*	*P*-value	Extent of nausea	*Z*	*P*-value
Sagittal cleaning Group A	1 (0–3)	0.047	0.962	2 (0–3.25)	− 0.639	0.523
Sagittal cleaning Group B	1 (0–3)			1 (0–3)		
Coronal cleaning Group A	1 (0–2.75)	0.179	0.858	1 (0–3)	− 1.297	0.195
Coronal cleaning Group B	1 (0–2)			2 (0–3)		

	Palate			Tongue		
	Time (s)	*t*	*P*-value	Time (s)	*t*	*P-*value
	X ± SD			X ± SD		
Sagittal cleaning	7.92 ± 3.02	− 2.116	0.036	6.68 ± 2.24	− 2.054	0.042
Coronal cleaning	7.46 ± 2.64			6.36 ± 2.22

**Table 5:** Stratified analysis of the extent of nausea caused by palate and tongue cleaning

Stratification factor	Palate				Tongue			
	Extent of nausea (sagittal)	Extent of nausea (coronal)	*Z*	*P*-value	Extent of nausea (sagittal)	Extent of nausea (coronal)	*Z*	*P*-value
*Sex*								
Male	1 (0–5)	2 (0–4)	− 0.336	0.737	2 (0–3)	1 (0–3)	− 1.588	0.112
Female	1 (0–2)	1 (0–2)	− 2.434	0.015	1 (0–3.5)	1 (0–3)	− 2.574	0.010
*Height*								
≥ 163.00 cm	1 (0–3)	1 (0–3)	− 1.036	0.300	2 (0–3)	1 (0–2)	− 3.121	0.002
< 163.00 cm	1 (0–2)	1 (0–2)	− 2.061	0.039	1 (0–4)	2 (0–4)	− 1.088	0.277
*BMI*								
Overweight and obese	1 (0–3)	1 (0–3)	− 0.427	0.669	1 (0–3)	1 (0–2)	− 1.681	0.093
Normal	1 (0–2)	1 (0–2)	− 1.984	0.047	2 (0–4)	1 (0–3)	− 2.416	0.016
Underweight	2 (1–4)	2 (1–3)	− 1.026	0.305	2 (0–4)	2 (0–3)	− 0.966	0.334

### Extent of nausea caused by two tongue cleaning directions

The scores for the extent of nausea elicited by cleaning the tongue coronally and sagittally were 1 (0–3) and 2 (0–3), respectively. The extent of nausea caused by sagittal cleaning was significantly higher than that caused by coronal cleaning (Wilcoxon rank-sum test: *Z* = − 2.990; *p* = 0.003). To rule out the influence of cleaning order, a randomized, crossover design was implemented, and the results indicate that the extent of nausea caused by cleaning the tongue was not influenced by the cleaning order (Table 4). The difference in the duration of tongue cleaning between the two cleaning directions was statistically, but not clinically, significant. Furthermore, the results of stratified analyses showed that the extent of nausea caused by sagittal cleaning was significantly higher among female participants, those with heights higher than medium, and participants with normal BMI (Table 5).

### Other discomforts reported by participants

The open-ended question about discomfort other than nausea was completed by 149 (54.0%) participants. Among the 149 responses listed, itching caused by cotton fibres (89.9%) was the most frequently reported discomfort. In addition, 41 (27.5%) participants complained that the 0.9% saline solution was too salty for them, while 7 (4.7%) participants felt that the solution was too cold.

## Discussion

The mean depths at which the palate and tongue could be cleaned without nausea were 6.75 ± 1.07 cm and 6.92 ± 1.11 cm, respectively, and the two were positively correlated (*t* = 0.730,* P* < 0.001), suggesting that, in clinical practice, the same cleaning depth can be used for both the tongue and palate of an individual patient. Further univariate analyses showed that sex was the main factor related to the tolerable depth of oral cleaning, with males able to tolerate deeper oral care, which may be attributable to innate differences in structural depths between the sexes [17, 18]. Similarly, Mimgu Park et al. reported that males had a longer depth of the gag reflex, which is consistent with our results [8]; the reason may be the longer maxillary arch size in males [19]. Therefore, men can tolerate deeper oral care. Hence, our study recommends that when performing oral care for adult males, the cleaning depth can be suitably increased.

Height was another factor that we expected to influence the tolerable cleaning depth; however, we did not detect any significant differences in the tolerable cleaning depths for either palate or tongue between height categories, and this factor failed to enter the regression equation, suggesting that the difference in cleaning depth between individuals of different heights was less than expected. Therefore, based on our study, we do not recommend that cleaning depth be changed according to patient height.

As shown in Tables 2 and 3, the association between BMI and cleaning depth was higher than expected, with BMI being a factor that significantly influenced tolerable cleaning depth. There is evidence that obese individuals have a greater risk of regurgitation and pulmonary aspiration than underweight patients [20]. Hence, based on our results, we recommend that the depth of cleaning of the tongue and palate should be increased for overweight/obese patients.

To reduce measurement errors, we tested tolerable cleaning depths for the tongue and palate three times. Unexpectedly, we found that tolerable cleaning depths for the tongue and palate gradually and significantly increased with the order of measurement (first to third), which could be related to increased tolerance of gagging reflexes in response to multiple stimuli. This finding suggests that multiple stimulation training could be used to increase tolerance depth in the clinic, particularly for patients with an overactive gag reflex. In addition, previous studies have proposed several useful methods for overcoming gag reflexes, including earplugs [21], relaxation, and distraction [22]. Moreover, some scholars have suggested that the ideal instruments for measuring the gag reflex should include the use of different materials and be applied with variable intensities, durations, and positions of stimuli [23]. One study used a standard disposable saliva ejector, with a stopper of heavy body addition silicone impression putty, as a device to measure the gag reflex depth of their participants [12]. Considering that the most common type of oral care equipment used in China is forceps with cotton balls [24], we used them for the measurement of cleaning depth in this investigation.

Our study compared the extent of nausea caused by coronal and sagittal cleaning (Fig. 1). The results show that the extent of nausea caused by sagittal cleaning was significantly higher than that caused by coronal cleaning, which is consistent with our clinical experience. This may be because, for coronal cleaning, the deep oropharynx is only accessed once, where sagittal cleaning requires repeated insertions into the deep oropharynx, and it is possible that depth is not adequately controlled. However, these differences were only significant among female participants and those with normal BMI, likely due to the sex ratio and BMI range of our participants; the proportions of female participants and those with normal BMI were 85% and 77%, respectively. Overall, based on our study, we recommend that the tongue and palate should be cleaned coronally.

When asked about types of discomfort other than nausea, subjects mentioned itching 134 times, with one research subject saying, "*The wool of the cotton ball passed through my oral mucosa, and it is truly itchy*!" Another research subject said, "*The cotton wool on the cotton ball hangs in the mouth, leaving so much fibre in my mouth*." These results demonstrate that cotton balls were not as comfortable as expected; thus, new materials and tools should be used to replace this approach. Gauze pads are widely used in Israel [25] and have been proven to help nurses implement more effective and gentle oral care [26]. In addition, a foam swab specifically designed for cleaning the tongue and palate has been reported in America; however, its cleaning effects have yet to be verified [11].

The second most commonly reported discomfort, following itching, was saltiness, which was mentioned 41 times. This indicates that the salinity of the 0.9% NaCl solution exceeds that of people’s daily diet and causes discomfort. Physiological saline has been recommended in textbooks for many years as a common oral care solution and is believed to contribute to oral cleansing and sterilization; however, there is scarce evidence to support its efficacy. With regard to safety, saline has no negative effects on patient oral mucosa [27]; therefore, the use of saline for oral care warrants further exploration.

In addition, a few participants mentioned coldness as a discomfort. This suggests that oral care at room temperature can be tolerated by most people; however, there are also some subjects sensitive to temperature. Some researchers in China have tried heated oral care solutions to improve comfort for patients during oral care, and they suggested that a specific temperature range could be selected by the patient according to their daily habits [28].

The strengths of this study include exploring the depth and direction of oral cleaning, which has not been researched before, providing valuable information for nurses to provide oral care using more scientific cleaning depth and direction.

### Limitation

Participants recruited in this study were healthy, and their average age was only 19.63 years. Due to the crucial roles of age and health in the gag reflex, the results of this study may not be representative of the overall situation for clinical patients. Further study is required to confirm these results in the clinic.

## Conclusion

This study found that the greatest tolerable cleaning depths for the palate and tongue, without causing nausea, were 6.75 ± 1.07 cm and 6.92 ± 1.11 cm, respectively. It is appropriate to increase these values when performing oral care for adult males and overweight/obese individuals. Multiple stimulation training is advised for patients with an overactive gag reflex to decrease their gag reflex sensitivity. The extent of nausea caused by sagittal cleaning was higher than that caused by coronal cleaning, and saline-soaked cotton balls can cause different kinds of discomfort. To improve oral care practice, further investigations should use gag reflex assessment tools and oral care standards with consideration of the health status of the patients.

## Data Availability

Data is available upon request. Contact e-mail: zhouyf@njmu.edu.cn.

## References

[1] Randall CL (2014). Gagging and its associations with dental care-related fear, fear of pain and beliefs about treatment. J Am Dent Assoc.

[2] Booker S, Murff S, Kitko L, Jablonski R (2013). Mouth care to reduce ventilator-associated pneumonia. Am J Nurs..

[3] Elad S, Raber-Durlacher JE, Brennan MT (2015). Basic oral care for hematology-oncology patients and hematopoietic stem cell transplantation recipients: a position paper from the joint task force of the Multinational Association of Supportive Care in Cancer/International Society of Oral Oncology (MASCC/ISOO) and the European Society for Blood and Marrow Transplantation (EBMT). Support Care Cancer..

[4] Liu C, Cao Y, Lin J, Ng L, Needleman I, Walsh T (2018). Oral care measures for preventing nursing home-acquired pneumonia. Cochrane Database Syst Rev..

[5] Pereira IF, Firmino RT, Meira HC, DO Egito Vasconcelos BC, DE Souza Noronha VRA, Santos VR (2019). Radiation-induced oral mucositis in Brazilian patients: prevalence and associated factors. In Vivo..

[6] El-Solh AA (2011). Association between pneumonia and oral care in nursing home residents. Lung..

[7] van Houtem CM, van Wijk AJ, Boomsma DI, Ligthart L, Visscher CM, de Jongh A (2015). Self-reported gagging in dentistry: prevalence, psycho-social correlates and oral health. J Oral Rehabil..

[8] Park M, Byun J, Jung J, Choi J (2020). The correlation of gagging threshold with intra-oral tactile and psychometric profiles in healthy subjects: a pilot study. J Oral Rehabil..

[9] Akarslan ZZ, Biçer AZ (2012). Utility of the gagging problem assessment questionnaire in assessing patient sensitivity to dental treatments. J Oral Rehabil..

[10] Matsui M, Chosa N, Shimoyama Y, Minami K, Kimura S, Kishi M (2014). Effects of tongue cleaning on bacterial flora in tongue coating and dental plaque: a crossover study. BMC Oral Health..

[11] Yates S, Gollins GJ. Oral care can be a matter of life and death. Drugs Today. https://www.americannursetoday.com/oral-care-can-be-a-matter-of-life-and-death/. Accessed Nov 2008.

[12] Karibe H, Okamoto A, Kato Y, Shimazu K, Goddard G (2018). Reliability, validity, and sex differences in a quantitative gag reflex measurement method. J Oral Rehabil..

[13] Östlund U (2011). Combining qualitative and quantitative research within mixed method research designs: a methodological review. Int J Nurs Stud..

[14] Li LM, Huang YQ. Clinical epidemiology. 2014.

[15] Imai K, Kitakoji H, Sakita M (2006). Gastric arrhythmia and nausea of motion sickness induced in healthy Japanese subjects viewing an optokinetic rotating drum. J Physiol Sci..

[16] Hsieh HF, Shannon SE (2005). Three approaches to qualitative content analysis. Qual Health Res..

[17] Louly F, Nouer PR, Janson G, Pinzan A (2011). Dental arch dimensions in the mixed dentition: a study of Brazilian children from 9 to 12 years of age. J Appl Oral Sci..

[18] Akkoç B, Arslan A, Kök H (2017). Automatic gender determination from 3D digital maxillary tooth plaster models based on the random forest algorithm and discrete cosine transform. Comput Methods Programs Biomed..

[19] Lee SJ, Moon SC, Kim TW, Nahm DS, Chang YI (2004). Tooth size and arch parameters of normal occlusion in a large korean sample. Korean J Orthod..

[20] Mahajan V, Hashmi J, Singh R, Samra T, Aneja S (2015). Comparative evaluation of gastric pH and volume in morbidly obese and lean patients undergoing elective surgery and effect of aspiration prophylaxis. J Clin Anesth..

[21] Cakmak YO, Ozdogmus O, Günay Y (2014). An earplug technique to reduce the gag reflex during dental procedures. Forsch Komplementmed..

[22] Elbay M, Tak Ö, Şermet Elbay Ü, Kaya C, Eryılmaz K (2016). The use of low-level laser therapy for controlling the gag reflex in children during intraoral radiography. Lasers Med Sci..

[23] van Linden van den Heuvell GF, de Boer B, Ter Pelkwijk BJ, Bildt MM, Stegenga B (2015). Gagging problem assessment: a re-evaluation. J Oral Rehabil..

[24] Qu X, Xie H, Zhang Q, Zhou X, Shi Z (2015). A survey on oral care practices for ventilator-assisted patients in intensive care units in 3A hospitals of mainland China. Int J Nurs Pract..

[25] DeKeyser Ganz F, Fink NF, Raanan O, Asher M, Bruttin M, Nun MB, Benbinishty J (2009). ICU nurses' oral-care practices and the current best evidence. J Nurs Scholarsh..

[26] Hitz Lindenmüller I, Lambrecht JT (2011). Oral care. Curr Probl Dermatol..

[27] Özden D, Türk G, Düger C, Güler EK, Tok F, Gülsoy Z (2014). Effects of oral care solutions on mucous membrane integrity and bacterial colonization. Nurs Crit Care..

[28] Zhou XQ, He YR (2014). The improvement of oral care fluid temperature in oral care application. Chinese Community Doctors..

